# A translational couch technique for total body irradiation

**DOI:** 10.1120/jacmp.v2i4.2597

**Published:** 2001-09-01

**Authors:** Mehrdad Sarfaraz, Cedric Yu, D. J. Chen, Leon Der

**Affiliations:** ^1^ Radiation Oncology Department University of Maryland Medical System 22 South Greene Street Baltimore Maryland 21201

**Keywords:** total body irradiation, translational couch, dose calculation, shielding blocks

## Abstract

We have constructed a computer controlled translational couch to administer total body irradiation reproducibly and safely. The system has replaced the previous stationary anterior‐posterior technique in our institution and 30 plus patients have been treated with it so far. In this technique, patients comfortably lie on a couch in supine and prone positions and are transported slowly through a narrow beam with the gantry in an upright position. Dose to the patient is determined by the couch velocity that is calculated based on physical parameters such as patient's dimensions, beam geometry, and machine dose rate. In our design, the couch velocity is continuously updated to compensate for machine dose rate fluctuations. The translational couch technique provides better dose uniformity within the patient compared to fixed beam techniques, and allows a more precise shielding block placement for organs at risk. At the same time, it presents a special challenge for dosimetry calculations. A dosimetry parameter is introduced that converts the moving beam output to the fixed beam output factor. Based on this factor, a simple dosimetry calculation method has been developed that takes advantage of conventional dosimetry parameters, eliminating extensive dosimetry measurements. Multiple point dose measurements within a phantom confirmed the validity of the calculation method.

PACS number(s): 87.53.–j, 87.66.–a

## INTRODUCTION

Total body irradiation (TBI) with photon beams is administered in radiation therapy centers for variety of the clinical situations with different techniques.^1–^, ^4^ The radiotherapy is usually combined with comprehensive chemotherapy either prior to or concurrent with the radiation. The radiation dose delivered throughout the patient body varies mainly due to the variation in patient thickness. However, it is desired to keep the dose nonuniformity within 10% of the prescription dose for best clinical results. Partial shielding for specific organs (e.g., lungs and kidneys) is often provided to minimize normal tissue complications.

The most common irradiation technique consists of anterior‐posterior fixed beams with the patient in standing position. This technique requires a source to surface distance (SSD) in excess of 3 m to encompass the patient within the large beams. The sickness and fatigue associated with chemotherapy makes it difficult for many patients to hold a standing position during the prolonged radiation time, resulting in poor reproducibility in setup. In an alternative technique, to alleviate patient discomfort to some extent and to overcome the limitation of treatment room dimensions, patients are treated in a semiseated position with bilateral beams. However, this technique suffers from poor dose uniformity and does not allow for effective shielding of the lungs and kidneys. Moreover, it is found that kidneys outlined on computed tomography (CT) scans show a dramatic shift in all directions during treatment in the above mentioned techniques,^5^ creating a serious shielding problem.

A few centers have employed a sweeping arc beam with patients lying on the floor under the machine.^6,^, ^7^ However, this technique requires utilization of a sophisticated custom‐made compensator for each patient. In another approach, a translational couch technique is utilized in which patients rest on a couch in supine and prone positions and are transported horizontally through a vertical beam.^8–^, ^11^ This technique presents a special challenge regarding dosimetry due to the moving beam dose delivery. Quest^11^ proposed a pragmatic calculation scheme for the moving beam TBI based on extensive data measurements for the specific treatment geometry. Gerig *et al* ^9^ introduced dynamic dosimetry parameters for moving beams and compared them to fixed beam parameters. We have theoretically derived the moving beam parameters and introduced a function that converts the moving beam output factor to a standard fixed beam output factor to simplify the calculations and measurements.

## MATERIALS AND METHODS

### A. Couch design and construction

The translational couch system consists of a steel frame, a moving cradle, and a driving assembly. Movement of the cradle is furnished by a stepping motor through two conveyor belts. An indexer controls the motor by sending it step pulses. The indexer is controlled by a set of commands, including one to move the couch with a calculated velocity. The dose to the patient is determined by the couch velocity assuming constant machine dose rate. The couch velocity is updated continuously based on the machine dose rate to compensate for any dose rate fluctuations. The linear accelerator is set to output the machine dose rate as an analog voltage signal, which is digitized by an analog‐to‐digital converter board. A rotary encoder and reader independently monitors the couch velocity for comparison with the calculated velocity. It warns the operator if there is more than 10% difference between two velocities and can stop the treatment at preset limits. All the boards are interfaced with a computer, which controls the treatment sequence.

### B. Treatment setup

**Figure 1:**
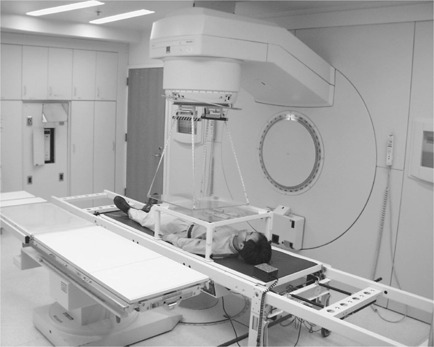
Translational couch TBI treatment setup.

Patients are transported through a vertical 6‐MV photons beam in supine and prone positions while lying on the couch. Source to couch distance is fixed at 160 cm, allowing 50‐cm clearance from the floor to eliminate undesirable backscatter radiation. Field width across the couch is set to its maximum of 40 cm at the isocenter, but field length along the couch movement may be set to any value from 5–40 cm at the isocenter. No immobilization device is utilized. Shielding blocks are placed on a tray that is supported by a table attached to the moving cradle. A 1‐cm plexiglass plate is hung from the gantry head close to the patient as the beam spoiler to increase the surface dose. The setup is pictured in Fig. 1.

The dose is prescribed to, and calculated for, the patient's midline at umbilicus, but it is also recorded at other body sections. In addition, dose limit to organs at risk is specified. The patient is brought in for simulation during which the SSD and thickness at several points for supine and prone positions are recorded. These parameters are entered into the computer along with the prescribed dose, field length, and selected machine dose rate for couch velocity calculation. Radiographic films are also taken at this time on which shielding blocks for lungs are drawn and constructed. A CT scan will be used to outline the kidneys for the block construction.

### C. Dosimetry

The tissue maximum ratios (TMR) were directly measured for the specific TBI treatment setup, out of concern over dependency of the TMR on the beam scatter condition at extended distances.^2^ The measurements of field size output factors and dosimetry verification were performed in a water tank (40×30×30 cm). The use of a phantom with dimensions close to a typical patient minimizes the errors in the determination of dose to the patient.^12^

The field size output factors were measured at an extended SSD for each collimator settings in three different phantom sizes (10×10×10, 20×20×20, and 40×30×30 cm) resembling legs, head, and torso of a typical patient.

A dose verification was performed with thermoluminescence dosimeters (TLD) placed inside an anthropomorphic phantom.

## THEORY

### A. Dose calculations

**Figure 2:**
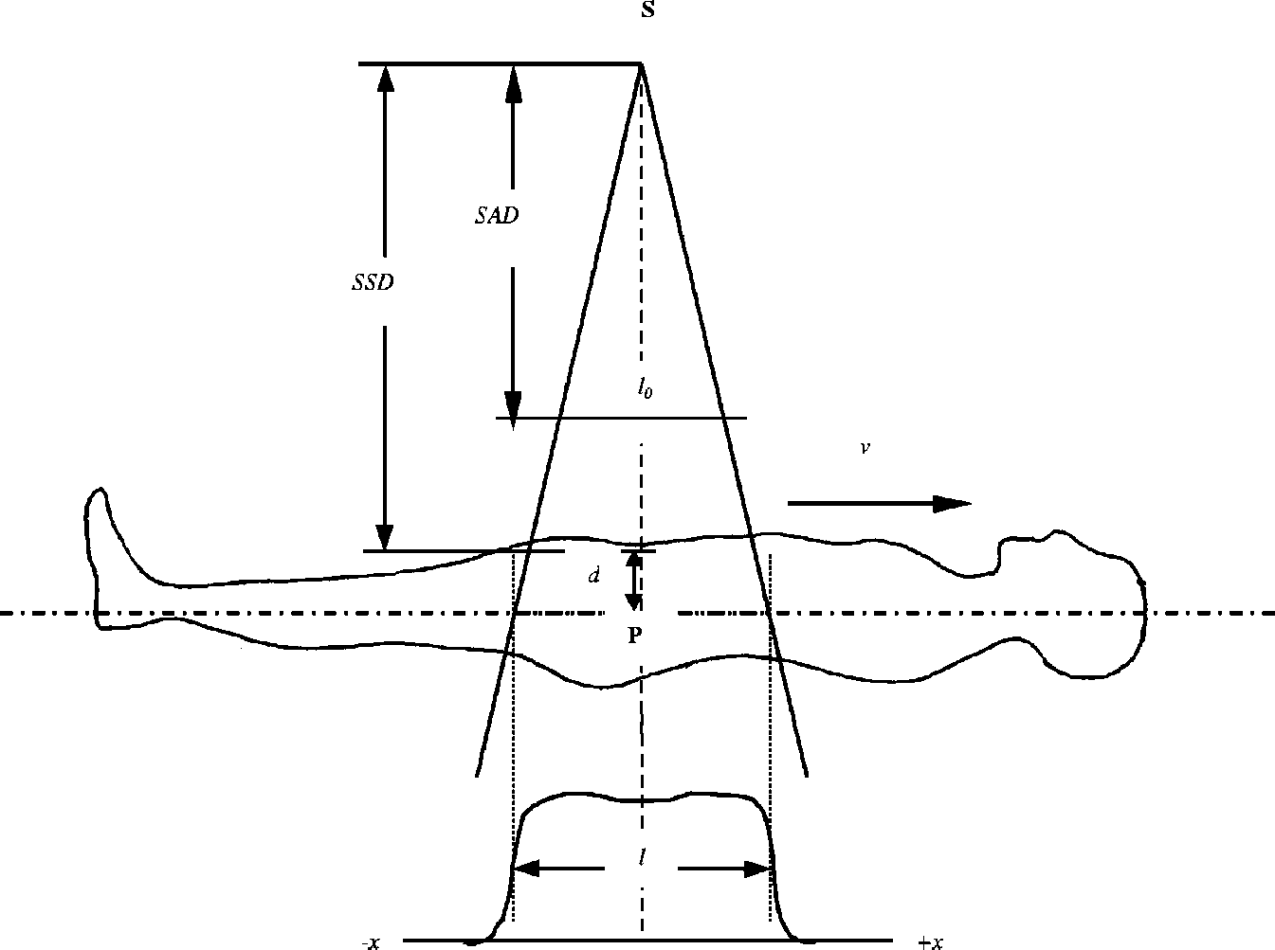
Notations used in deriving dosimetry relations in the text.

In the translational couch irradiation, every point within the patient sweeps the entire longitudinal beam profile as it moves under the field. Referring to Fig. 2, the instantaneous dose rate to point *P* at depth *d*, for equivalent field size *r* corresponding to field width *w* and length *l* can be written as

(1) $$\dot{D}(d,x,r)={\dot{D}}_{\text{CAX}}(d,r)\times OAR(d,x,l),$$

where ${\dot{D}}_{\text{CAX}}$ is the dose rate on the central axis of the beam and *OAR* is the off‐axis ratio at distance *x* off the beam axis. The total dose at *P* can be calculated by integration of the instantaneous dose rate over time as

(2) $$D(d,r)=\int_{-\infty }^{+\infty }\dot{D}(d,x,r)dt=\int_{-\infty }^{+\infty }{\dot{D}}_{\text{CAX}}(d,r)\times OAR(d,x,l)dt.$$

For the couch moving at constant velocity *v*, x=υt thus dx=υdt. Substituting time *t* by *x* in Eq. (2) and assuming machine dose rate on central axis is constant (machine dose rate fluctuations is compensated by the firmware as described within the text) results to

(3) $$D(d,r)={\dot{D}}_{\text{CAX}}(d,x)\times \frac{1}{\upsilon }\times \int_{-\infty }^{+\infty }OAR(d,x,l)dx.$$

The ratio of the dose at depth *d* divided by the dose at depth $d_{\max}$ is defined as the moving beam *TMR*, similar to the fixed beam *TMR*, and can be obtained from Eq. (3),

(4) $$TMR_{\text{moving}}(d,r)=\frac{D(d,r)}{D(d_{\max},r)}=\frac{{\dot{D}}_{\text{CAX}}(d,r)\times \int_{-\infty }^{+\infty }OAR(d,x,l)dx}{{\dot{D}}_{\text{CAX}}(d_{\max},r)\times \int_{-\infty }^{+\infty }OAR(d_{\max},x,l)dx}$$

or

(5) $$TMR_{\text{moving}}(d,r)=TMR_{\text{fixed}}(d,r)\frac{\int_{-\infty }^{+\infty }OAR(d,x,l)dx}{\int_{-\infty }^{+\infty }OAR(d_{\max},x,l)dx}.$$

Therefore the dose at any depth can be calculated from the dose at $d_{\max}$ knowing the $TMR_{\text{moving}}$,

(6) $$D(d,r)=D(d_{\max},r)\times TMR_{\text{moving}}(d,r).$$

### B. Couch velocity

The total dose at point *P* in the patient in Eq. (6) can be expanded using Eq. (3) as

(7) $$D(d,r)={\dot{D}}_{\text{CAX}}(d_{\max},r)\times TMR_{\text{moving}}(d,r)\times \frac{1}{\upsilon }\times \int_{-\infty }^{+\infty }OAR(d_{\max},x,l)dx,$$

where dose on the central axis at depth $d_{\max}$ at extended SSD can be derived from

(8) $${\dot{D}}_{\text{CAX}}(d_{\max},r)=\dot{M}\times D_{0}\times S_{c,p}\times TF\times {\left(\frac{100}{\text{SSD}+d}\right)}^{2}.$$

In the above equations, $\dot{M}$ is the machine dose rate at the isocenter, $D_{0}$ is the reference calibration dose at the isocenter and depth $d_{\max}$, $S_{c,p}$ is field size output factor at extended SSD, and *T.F.* is the spoiler‐tray factor.

Denoting

(9) $$m=\frac{\int_{-\infty }^{+\infty }OAR(d_{\max},x,l)}{l}$$

and noting that

$$l=l_{0}\times \left(\frac{\text{SSD}+d}{100}\right),$$

the total dose received at point *P*, using Eqs. (7) to (9) can be expressed as

(10) $$D(d,r)=\dot{M}\times D_{0}\times TMR_{\text{moving}}(d,r)\times S_{c,p}\times TF\times \frac{m\times l_{0}}{\upsilon }\times \left(\frac{100}{\text{SSD}+d}\right).$$

It can be seen from last term in Eq. (10) that for the moving beam, dose is inversely proportional to distance from source, unlike the stationary beam that is proportional to the inverse square of the distance from source. The *M* factor in the above equations is calculated as the area under the beam profile at depth $d_{\max}$, normalized to the field length. Defining *M* in this way makes the dependency of the dose on inverse distance from source explicit in Eq. (10).

Rearranging Eq. (10), the couch velocity for the prescribed dose *PD* at depth *d* for field size *r* can be calculated from

(11) $$\upsilon =\frac{\dot{M}\times D_{0}\times TMR_{\text{moving}}(d,r)\times S_{c,p}\times TF\times m\times l_{0}\times (100/\text{SSD}+d)}{PD}$$

The corresponding total treatment time *t* for a patient height *L*, turning the beam on l/2 before and after patient moves inside and outside of the beam will be

(12) $$T=\frac{L+l}{\upsilon }.$$

## RESULTS

### C. Moving and fixed TMRs

**Figure 3:**
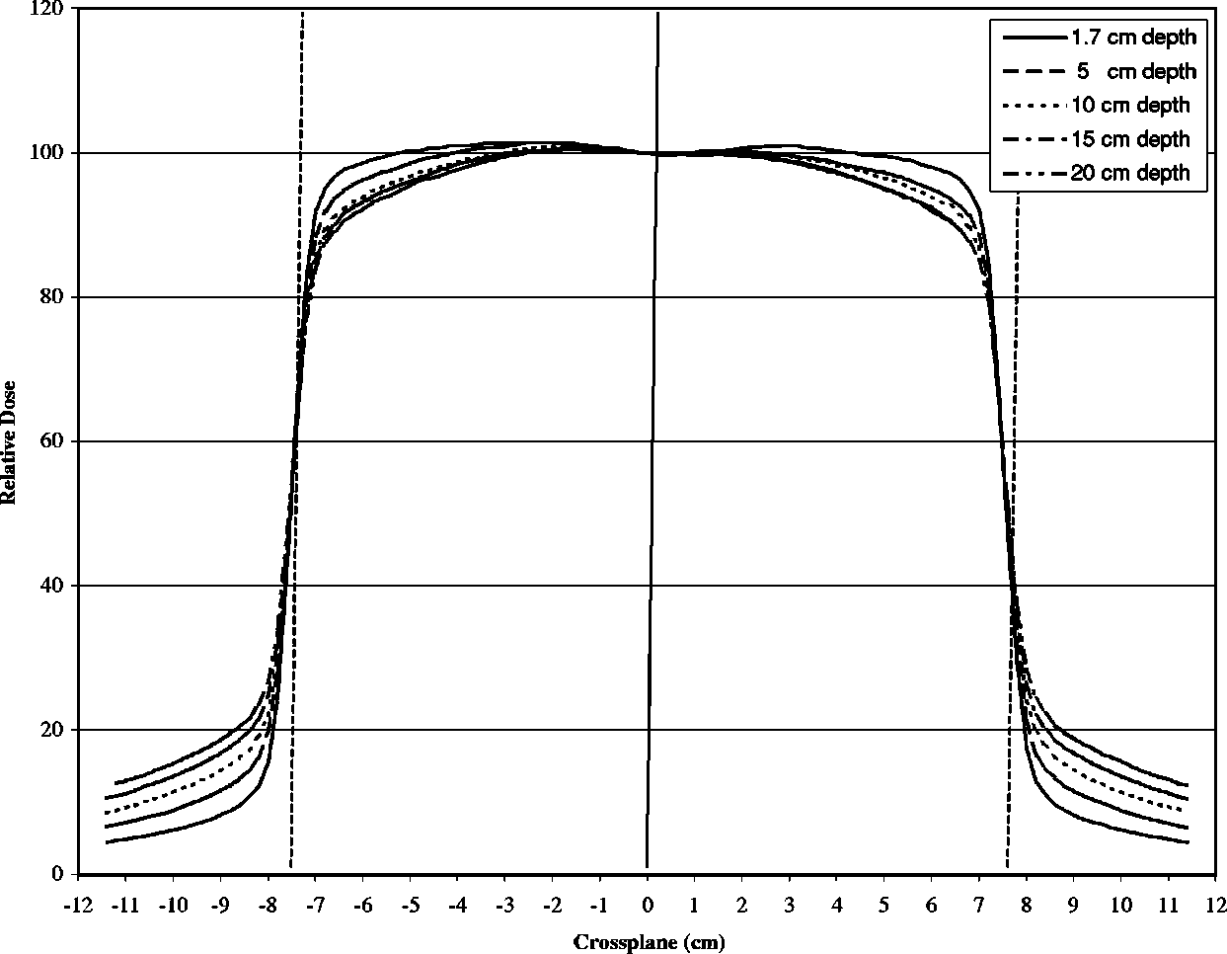
Beam profiles at the isocenter for 6‐MV photons, 15×15 cm field size for different depths. The profiles are all normalized to central axis.

Equation (5) indicates that the moving and fixed TMRs can be different because the area under the normalized beam profile at depth *d* may be different from same area at depth $d_{\max}$. However, moving and fixed beams TMRs directly measured at certain depths and field sizes on our treatment machine proved to have the same values. The reason for this effect can be found in the shape of the normalized beam profiles in Fig. 3. It can be intuitively seen that the faster dose falloff on shoulders of the beam profiles at higher depths can be compensated by a slower dose falloff on tail regions, resulting in the same area under the profiles. The only exception was in the buildup region, where profiles feature distinct “horns” at the off‐axis. Therefore, in our case it was sufficient to measure only the fixed TMR. The situation might be different for different treatment machines and the issue should be examined independently by other investigators.

### D. Output factors

The dose rate at an extended distance for the moving beam is related to fixed beam and standard calibration geometry by Eqs. (8) and (10). In fact, from Eq. (10), it can be inferred that the only factor that converts moving beam output to fixed beam output for the same setup is the *M* factor. The *M* factor, designated as the moving beam output factor, can be considered as the ratio of the area under the actual beam profile to the area under an idealized beam profile, where dose is 100% of the central axis over the field length and zero elsewhere. It can either be calculated from Eq. (9) or measured as the ratio of the integral dose with the moving beam divided by the stationary beam central axis dose for the same setup. Its value for our machine was found to be 1.05.

The dependency of the dose rate in phantom on inverse square distance from the source was examined by measuring doses for 100 and 150 cm from the source for 10×10 cm field size. It was found that the inverse square relation estimates dose along the central axis to better than 0.5% for the reference field size and depth. The change in dose rate at an extended SSD due to the change in the collimator setting and phantom sizes is accounted for by the measured field size output factors.

### E. Effect of the beam spoiler

Different thickness Lucite sheets at various distances from the surface were studied as the beam spoiler. It was found that a 1‐cm thick Lucite sheet placed 15 cm from the surface raises skin dose from 50% to 87% without changing beam characteristics at deeper depths. It is estimated that total skin dose from the opposite fields would be larger than 95% of the prescribed dose at the midline of a typical patient with this beam spoiler.

### F. Shielding block

**Figure 4:**
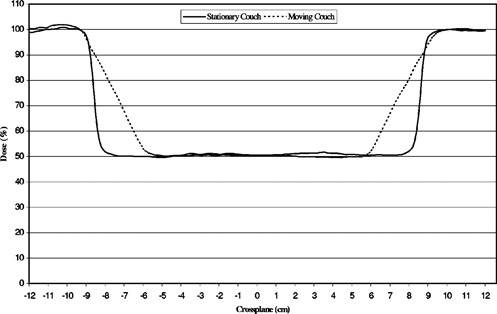
Dose profiles under the shielding block in stationary and moving beams.

In order to compare stationary and moving beams shielding geometries, films were placed at a 8‐cm depth in “solid water” blocks, 158 cm from the source, and exposed with a partial cerrobend block in a 40×20 cm field. The Cerrobend block was 1.8‐cm thick, 15‐cm long in the direction of motion, and was placed 8 cm above the surface. The shielded area dose profiles in the direction of couch movement for stationary and moving beams are shown in Fig. 4.

It can be seen that dose decreases from 100% to 50% over 3 cm near the block edge for the moving beam, compared to less than 0.5 cm for the stationary beam. This is due to the fact that points near block edge receive primary unattenuated photons before beam moves over the block. The magnitude of this effect depends on beam divergence and can be minimized by reducing the block to skin distance or decreasing field width.

### G. Dose verification

**Figure 5:**
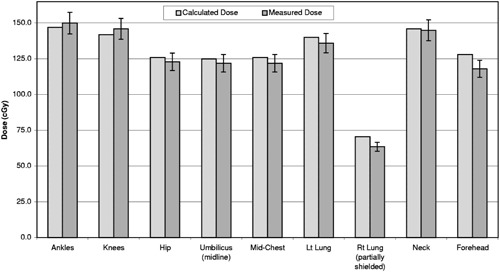
Comparison of calculated and measured doses in midplane of an anthropomorphic phantom for the translational couch TBI. The error bars represent one standard deviation of the measurements.

The anthropomorphic phantom was irradiated to deliver 125 cGy at the midline of the umbilicus level. The dose to midline of the right lung, not corrected for inhomogeneity, was to be 62.5 cGy. The expected doses and TLD measured doses are shown in Fig. 5. The agreement between expected and measured doses were better than 3% for all sites except forehead and shielded right lung. The forehead dose was measured 7.8% lower than calculated. This is thought to be mainly due to the lack of full scatter condition near top of the head. Points within one field width from the top of the head and toes of the feet do not receive full scattered photons from the portion of the beam that falls in air. Fortunately, this effect works in favor of dose uniformity within patient because doses at these points are normally higher than the prescribed dose at CAX. The right lung dose was 10% lower than calculated. It should be noted that our inhomogeneity calculation method is a simple effective depth method and does not account for change in the scatter condition due to the presence of lung inside and outside of the calculation plane. The longitudinal doses at the patient midline with respect to umbilicus varied from 100% at hip and mid‐chest, to 117% at neck and ankles for the phantom.

**Figure 6:**
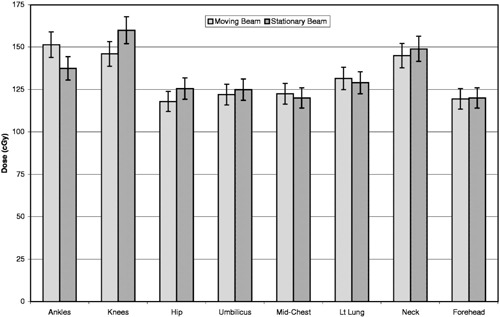
Comparison of the dose variations in the anthropomorphic phantom for translational couch and stationary TBI techniques. The error bars represent one standard deviation of the measurements.

Dose variations can be compared with the previous stationary beam technique without utilization of compensators in this institution. As it can be seen from Fig. 6, dose uniformity was slightly better with the moving beam technique.

## DISCUSSION

We have implemented a safe and reproducible TBI technique with increased dose uniformity within the patient. The moving beam exhibits less dose variations in the patient's longitudinal axis than fixed beam techniques because each point within the patient averages the entire beam profile in contrast to the fixed beam that points are under one point of the profile at all times. In addition, a closer relation between peripheral and midline doses is expected, compared with stationary beams because dose is a function of the inverse SSD rather than the inverse square. There is a potential for achieving even higher dose uniformity in this technique by couch velocity modulation based on patient thickness variation.^13^

This technique allows a precise shielding placement for organs at risk. However, beam divergence and couch movement creates a wide beam penumbra along the couch movement. The magnitude of this effect depends on beam divergence and can be minimized by reducing the block to skin distance or decreasing field width. A moving block tray is proposed to optimally shield the critical structure, while minimizing the penumbra in the moving beam TBI.^14^ Meanwhile, physicians are advised to be generous while drawing the blocks in the couch movement direction.

For a typical patient of 30‐cm thick and 200‐cm tall treated for 62.5 cGy per beam at 150 SAD and 15×40 cm field size, couch velocity would be 10 cm/min if we keep the dose rate within the patient the same as our stationary beam technique, i.e., 30 cGy/min. The corresponding treatment time is 20 min per beam, nearly twice the time for the stationary beam technique. To integrate this technique into a routine clinical schedule without inconveniencing personnel, treatment time should be at least reduced by half. This can be easily achieved by either increasing the machine dose rate, thus accepting increase in dose rate within patient, or increasing longitudinal field size. Larger field sizes are not preferred because they create wider shielding block penumbra as was explained previously. In addition, compensating for patient thickness variations by velocity modulation in a later stage can be more effective with smaller field sizes. Investigation is underway to possibly increase the dose rate within the patient.

The translational couch dose delivery demands quality assurance tests in addition to those that are normally performed on treatment machine. The moving output factor, *m*, must be checked periodically by measuring the ratio of stationary to moving doses for the same geometry. This factor will monitor any changes in the system, including the beam profile and the moving couch mechanism.

## ACKNOWLEDGMENTS

Authors would like to acknowledge Vincent Frouhar and Jack Ames for initiation of this project. We would like to thank J. Szanto and P. Genest from the Ottawa Regional Cancer Center, Canada for sharing with us their experience with the moving couch TBI. Also, we thank Thomas Holmes for reviewing the manuscript and offering valuable comments.
